# Artificial intelligence applications facilitate decision-making in cataract surgery for highly myopic patients

**DOI:** 10.3389/fcell.2025.1613634

**Published:** 2025-09-04

**Authors:** Kaimeng Su, Wenwen He, Haifeng Jiang, Keke Zhang, Jiao Qi, Jiaqi Meng, Yu Du, Kaiwen Cheng, Xiaoxin Hu, Dongling Guo, Haike Guo, Yong Wang, Yi Lu, Xiangjia Zhu

**Affiliations:** ^1^ Eye Institute and Department of Ophthalmology, Eye and ENT Hospital, Fudan University, Shanghai, China; ^2^ NHC Key Laboratory of Myopia and Related Eye Diseases; Key Laboratory of Myopia and Related Eye Diseases, Chinese Academy of Medical Sciences, Shanghai, China; ^3^ Shanghai Key Laboratory of Visual Impairment and Restoration, Shanghai, China; ^4^ Aier Eye Hospital of Wuhan University (Wuhan Aier Eye Hospital), Wuhan, Hubei Province, China; ^5^ Department of Ophthalmology, Shanghai Heping Eye Hospital, Shanghai, China; ^6^ State Key Laboratory of Medical Neurobiology, Fudan University, Shanghai, China

**Keywords:** machine learning, surgical decision-making, high myopia, cataract, artificial intelligence

## Abstract

**Background:**

Surgical decision-making for highly myopic cataracts requires a high level of expertise. We, therefore, aimed to develop a preliminary artificial intelligence (AI) model for surgical decision-making in highly myopic cataracts, based on previous deep learning models.

**Materials and methods:**

We first established a highly myopic cataract decision-making AI model by integrating cataract grading and postoperative visual acuity prediction models of highly myopic eyes, which we had developed previously, with surgical decision logic. The outcomes of surgical decision-making were classified into four categories: surgery not advised, cataract surgery recommended, retinal surgery recommended, and combined cataract–retinal surgery recommended. The gold standard for surgical decision is defined as the decision jointly made by two professional ophthalmologists together (X.Z. and Y.W.). If the decision-makings regarding highly myopic cataract surgery were not fully consistent, a final judgment was made by a third expert (Y.L.). Subsequently, we evaluated the accuracy of AI model’s surgical decision-making against the gold standard and doctors at different levels, using both internal (107 highly myopic eyes from Eye and ENT Hospital, Fudan University) and external (55 highly myopic eyes from Wuhan Aier Eye Hospital) test datasets.

**Results:**

In the internal and external datasets, according to the Lens Opacities Classification System (LOCS) III international standards for cataract grading, 99.07% and 87.27% of automatic nuclear grading, along with 88.79% and 61.82% of automatic cortical grading, respectively, had an absolute prediction error of ≤1.0 compared with the gold standard. The mean postoperative visual acuity prediction error was 0.1560 and 0.3057 logMAR in the internal and external datasets, respectively. Finally, the consistency of the AI model’s surgical decisions with the gold standard for highly myopic cataract patients in the internal and external datasets was 96.26% and 81.82%, respectively. AI demonstrated substantial agreement with the gold standard (Kappa value = 0.811 and 0.556 in the internal and external datasets, respectively).

**Conclusion:**

The AI decision-making model for highly myopic cataracts, based on two deep learning models, demonstrated good performance and may assist doctors in complex surgical decision-making for highly myopic cataracts.

## Introduction

Cataracts are the primary cause of reversible vision loss in the global elderly population (Imelda et al., 2022; Ruiss et al., 2022; Jiang et al., 2023). In 2020, approximately 15 million people over the age of 50 suffered from cataract-induced blindness (Cicinelli et al., 2023). Moreover, high myopia, an important factor for cataracts (Tan et al., 2018; Swierczynska et al., 2025; Wei et al., 2025), currently affects approximately 277 million individuals globally (Chen et al., 2024), and this number continues to increase (Nakao et al., 2021; Tsai et al., 2021; Swierczynska et al., 2025). The global aging trend and the increasing prevalence of high myopia suggest a further expansion of the global population of highly myopic cataract patients (Jiang et al., 2023). Therefore, the management of highly myopic cataracts is expected to become a key part of future ophthalmic care.

As surgery is currently the only treatment for cataracts, accurate surgical decision-making has become crucial for the management of highly myopic cataracts (Nakao et al., 2021; Tavassoli et al., 2024). It is worth mentioning that highly myopic cataracts are often accompanied by complex retinal conditions (Haarman et al., 2022; Hopf et al., 2022; Carla et al., 2025) and are usually combined with various vision-affected eye diseases, such as epiretinal traction, macular retinoschisis, retinal thickening, lamellar hole (Panozzo and Mercanti, 2004), foveal retinoschisis (Takano and Kishi, 1999), foveal retinal detachment (Takano and Kishi, 1999; Baba et al., 2003), and choroidal neovascularization (CNV) (Cheung et al., 2017; Kumar et al., 2021; Yao et al., 2021; Zhang et al., 2023). Therefore, surgical decisions for highly myopic cataracts are usually more complex than those for general cataracts. Opportunities exist to strengthen diagnostic capacity for highly myopic cataracts in resource-constrained primary care settings worldwide, or the lack of diagnostic capacity may lead to increasing cataract surgical risks and cause a large number of cases to be concentrated in tertiary hospitals, further resulting in insufficient distribution of medical resources (Keel et al., 2021). As a result, it is essential to develop an artificial intelligence (AI) model for highly myopic cataract surgical decision-making in the future.

By integrating an existing automatic cataract grading model and a high-myopia cataract postoperative best-corrected visual acuity (BCVA) prediction algorithm, this study aims to develop a possible AI model for highly myopic cataract surgical decision-making (Wei et al., 2021; Lu et al., 2022). The model was validated based on the internal and external test datasets through comparative analysis with manual surgical decision-making to evaluate its present accuracy. It aims to establish a foundation for the development of accurate AI models for surgical decision-making in highly myopic cataract cases in the future while currently offering surgical decision-making guidance for less-experienced doctors in managing complex cases.

## Materials and methods

### Ethics statement

This study was approved by the Institutional Review Board of the Eye and Ear, Nose, and Throat (EENT) Hospital of Fudan University (Shanghai, China). All procedures were conducted in accordance with the principles of the Declaration of Helsinki and the approved protocol. Clinical trial registration: NCT03062085 (www.clinicaltrials.gov).

### Case source

This AI model for highly myopic cataract surgical decision-making comprised an internal test dataset and an external test dataset. The internal dataset contained 107 cases sourced from the Department of Ophthalmology, EENT Hospital, Fudan University (from January 2023 to December 2023). The inclusion criteria for patients were as follows: (1) cataract patients with axial length (AL) of > 26.0 mm, (2) preoperative cataract cases with reliable macular OCT measurements, and (3) cataract patients with a record of preoperative BCVA and postoperative BCVA at 4 weeks after cataract surgery. Exclusion criteria were as follows: (1) corneal opacity or other corneal pathologies potentially compromising the visual pathway, (2) congenital ocular abnormalities, (3) neuropathic conditions affecting visual acuity, (4) ocular trauma, and (5) eyes with not assessable cataract status due to poor fixation, insufficient pupil dilation, or obscured observation areas. Another external dataset contained 55 cases sourced from the ophthalmic database of Wuhan Aier Eye Hospital (from January 2023 to December 2023) with identical inclusion and exclusion criteria to the internal dataset.

### Data collection

The test dataset for this AI model included actual preoperative visual acuities, postoperative visual acuities, and the axial length data recorded in hospital systems and imaging resources.

Imaging resources consisted of slit-lamp photographs and OCT scans of highly myopic cataract-affected eyes. For slit-lamp anterior segment imaging of cataract eyes, slit-beam and diffuse illumination photographs were captured using illumination and viewing arms positioned at a 30-degree angle relative to each other, whereas retroillumination photographs were focused on the posterior capsule. Distinct imaging modes were used for specific cataract subtypes: slit-beam mode for nuclear cataracts, diffuse-illumination mode for cortical cataracts, and retroillumination mode for posterior subcapsular cataracts. All ocular photographs were acquired under mydriatic conditions. OCT images were obtained using the Spectralis OCT system (Heidelberg Engineering, Germany) and Cirrus OCT (Carl Zeiss Meditec, United States) in the internal dataset, while the OCT images were obtained using the DRI OCT Triton (Topcon, Japan) and Rtvue XR (Optovue, Germany) in the external dataset.

### Cataract identification and diagnosis

The automatic cataract grading model used an advanced deep learning architecture, with all slit-lamp photographs undergoing normalization before model input. The model first performed capture mode identification to differentiate nuclear, cortical, and posterior subcapsular cataracts, followed by lesion localization using Faster R-CNN for region-of-interest (ROI) detection and cataract severity prediction via ResNet-101. Grading adhered to the Lens Opacities Classification System (LOCS) III international standards: nuclear cataracts were classified from 1.0 to 6.0 based on nuclear color, while cortical and posterior subcapsular cataracts were graded from 1.0 to 5.0 based on transparency. This architecture enabled automated classification of all three cataract types. Training, validation, and testing processes were detailed in prior publications (Lu et al., 2022).

The manual cataract grading was independently performed by doctors with varying expertise (K.Z., J.Q., and X.H. listed in descending experience order). The grading results of doctors were divided into three levels based on experience: K.Z. as the senior doctor, J.Q. as the junior doctor, and X.H. as the resident. The gold standard for cataract grading is defined as the decision jointly made by two professional ophthalmologists (X.Z. and Y.W.). If the surgical decision-making regarding highly myopic cataracts was not fully consistent, a final judgment was made by another expert (Y.L.). In this study, junior doctor refers to an attending ophthalmologist, whereas senior doctor represents an ophthalmologist of higher rank beyond the attending level. During this study, all doctors were blinded to the results of other doctors and those of the AI model.

### Postoperative visual acuity prediction in highly myopic cataract-affected eyes

The postoperative visual acuity prediction model for highly myopic cataract-affected eyes employed a deep learning framework. All input OCT photographs were highly normalized prior to processing. The model uses five different deep convolutional neural network (CNN) algorithms to construct an ensemble learning, including 18, 34, 50, and 101 layers of deep residual learning image recognition (ResNet, Microsoft Research) (ResNet-18, ResNet-34, ResNet-50, and ResNet-101) and Inception v3. Through this ensemble learning, this model was able to predict postoperative vision outcomes in eyes affected by high myopia-related cataracts. The specific training, validation, and testing processes can be found in the previous report (Wei et al., 2021).

The data on actual visual acuities after operations were sourced from the internal and external datasets, primarily from the clinical records in both hospitals.

### Highly myopic cataract surgical decision-making

This AI-based surgical decision-making model for highly myopic cataracts integrated two previously published models: the automatic cataract grading model and the postoperative visual acuity prediction model, synthesizing cataract severity assessment, postoperative visual improvement potential, and surgical risks to generate surgical decisions. AI surgical decisions were categorized into four categories: 0 (surgery not advised), 1 (cataract surgery recommended), 2 (retinal surgery recommended), and 3 (combined cataract–retinal surgery recommended).

After data are input into the AI decision-making model, the surgical decision results can be obtained in at most five steps. The first step is the judgment of preoperative visual acuity; the second step is the judgment of postoperative visual acuity improvement; the third step is the calculation and judgment of automatic cataract grading results; the fourth step is the judgment of OCT photos; and the fifth step is the judgment of the axial length. The logic of the AI-based decision-making process for highly myopic cataracts is detailed in Supplementary Figure S1. Manual surgical decision-making was independently performed by doctors with varying expertise (senior, junior, and resident, as mentioned above). The methodology for defining the gold standard followed the aforementioned procedures.

### Performance validation and statistical analysis

#### Cataract grading performance

This study first reevaluated the cataract automatic grading model’s performance for nuclear and cortical cataracts using internal (n = 107) and external (n = 55) datasets. To assess grading accuracy, differences between AI-predicted grades and the gold standard predictions were analyzed according to LOCS Ⅲ, with the absolute difference between the predicted grades from the AI model and the gold standard defined as the grading prediction errors and the percentage of grading prediction errors of ≤1.0 defined as R_e1.0_ (Lu et al., 2022). The intraclass correlation coefficient (ICC) was calculated between the AI model and the standard to analyze diagnostic performance. Additionally, diagnostic performance was evaluated using the following indices: accuracy = (true positive + true negative)/(true positive + true negative + false positive + false negative); sensitivity = true positive/(true positive + false negative); specificity = true negative/(true negative + false positive). True-positive, true-negative, false-positive, and false-negative values were defined according to the surgical decision based on nuclear and cortical cataract grading (if the nuclear cataract grade is >3.5 or the cortical cataract grade is >3.5, then cataract surgery is recommended for the eye). Receiver operating characteristic (ROC) curves were plotted, with area under the curve (AUC) calculated using the pROC package and compared using DeLong’s test (statistical significance: *p* < 0.05) (Lu et al., 2022).

#### Performance of postoperative visual acuity prediction

This study also analyzed the performance of postoperative visual acuity prediction algorithms in internal (n = 107) and external datasets (n = 55). The BCVA at 4 weeks after cataract surgery was considered the ground truth. The Snellen VA was converted to the logarithm of minimal angle of resolution (logMAR) as described previously, while counting fingers, hand motion, light perception, and no light perception were assigned a value of 1.9, 2.3, 2.7, and 3.0, respectively (Lange et al., 2009). Two groups were formed based on the actual BCVA values; the good VA group included eyes with actual BCVA values <0.30 logMAR (Snellen 6/12 or higher), whereas the poor VA group included eyes with actual BCVA values >0.30 logMAR (Snellen 6/12 or lower). To evaluate the differences in logMAR postoperative BCVA between the prediction and ground truth, we calculated the mean absolute error (MAE) and the root mean square error (RMSE). Then, the percentage of BCVA prediction errors within ±0.30 logMAR (Snellen 6/12, R_e0.30logMAR_) was calculated. The definitions of MAE, RMSE, and R_e0.30logMAR_ were detailed in previous research on the postoperative visual acuity prediction model (Wei et al., 2021).

#### Highly myopic cataract surgical decision-making performance

The accuracy of the AI-based surgical decision-making model for highly myopic cataracts was validated using the internal (n = 107) and external (n = 55) datasets. The consistency between the decision-making performance of this AI model and the gold standard was quantified. In addition, the Kappa values of the AI model’s surgical decision-making compared to the gold standard were calculated. Similar analyses were conducted to evaluate the consistency and the Kappa value between doctors of varying experience levels (senior doctor, junior doctor, and resident) and the gold standard. The gold standard was established as described above. Additionally, we also analyzed the performance of the model and doctors (with varying experience: senior doctor, junior doctor, and the resident) on special cases compared to the gold standard’s performance. Special cases were defined as eyes that required no surgery, retinal surgery, or combined retinal and cataract surgery. Heatmaps visualized the consistency between the AI model and the gold standard, along with each doctor (the senior doctor, the junior doctor, and the resident) in both internal and external datasets.

All analyses were conducted in R software (version 4.4.2) and SPSS Statistics 20.0 (Lu et al., 2022). A *p*-value of less than 0.05 was considered statistically significant.

## Results

### Cataract grading performance

A total of 107 slit-lamp photographs of eyes from the internal database and 55 slit-lamp photographs of eyes from the external database were used to test the AI model’s cataract grading performance.

**FIGURE 1:**
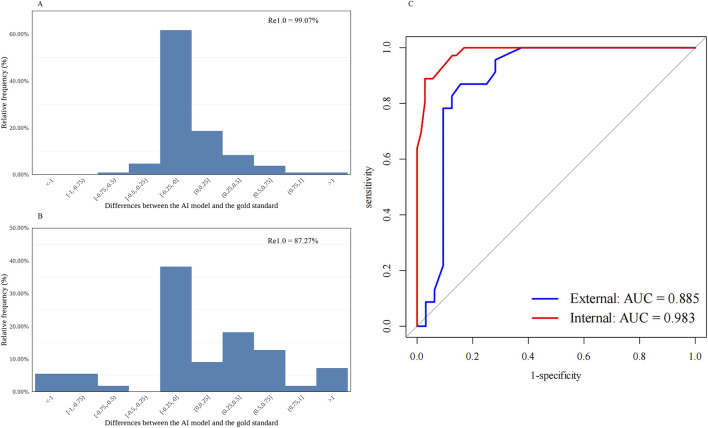
Distribution of the differences between the AI-predicted and the standard values and the receiver operating characteristic curve for automatic nuclear cataract grading. **(A)** Distribution of the differences in the internal dataset (n = 107). **(B)** Distribution of the differences in the external dataset (n = 55). **(C)** Receiver operating characteristic curves and areas under the curves: internal = 0.983; external = 0.885. AI, artificial intelligence; R_e1.0_, the percentage of cataract grading absolute prediction errors of ≤1.0; AUC, areas under the curves.

**FIGURE 2:**
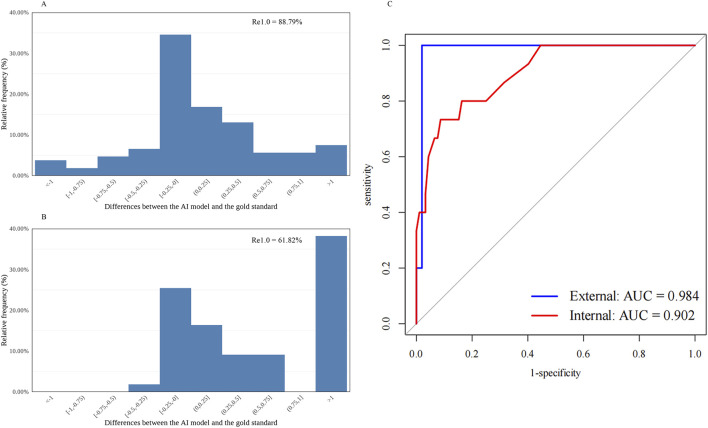
Distribution of the differences between the AI-predicted and the standard values and the receiver operating characteristic curve for automatic cortical cataract grading. **(A)** Distribution of the differences in the internal dataset (n = 107). **(B)** Distribution of the differences in the external dataset (n = 55). **(C)** Receiver operating characteristic curves and areas under the curves: internal = 0.902; external = 0.984. AI, artificial intelligence; R_e1.0_, the percentage of cataract grading absolute prediction errors of ≤1.0; AUC, areas under the curves.

The test results showed good consistency in the AI model’s performance on nuclear and cortical cataract grading. For nuclear cataract grading, 99.07% (106/107, internal) and 87.27% (48/55, external) of slit-beam photographs had an absolute prediction error of ≤ 1.0 (Table 1; Figures 1A,B). The R_e1.0_ values of cortical cataract grading were 88.79% (95/107, internal) and 61.82% (34/55, external) (Table 1; Figures 2A,B).

**TABLE 1:** Summary statistics for grading performance of the cataract AI program in the internal (n = 107) and external datasets (n = 55).

Parameters	Nuclear cataract		Cortical cataract	
	Internal	External	Internal	External
Re1.0 (%)	99.07% (106/107)	87.27% (48/55)	88.79% (95/107)	61.82% (34/55)
ICC between standard and automatic (%)	0.962	0.796	0.780	0.360
Accuracy (%)	92.52%	85.45%	89.72%	96.36%
Sensitivity (%)	88.89%	82.61%	66.67%	100.00%
Specificity (%)	94.37%	87.50%	93.48%	96.00%
AUC	0.983	0.885	0.902	0.984

AI, artificial intelligence; AUC, area under the curve; ICC, intraclass correlation coefficient; Re1.0, the percentage of cataract grading absolute prediction errors of ≤1.0; accuracy = (true positive + true negative)/(true positive + true negative + false positive + false negative); sensitivity = true positive/(true positive + false negative); specificity = true negative/(true negative + false positive).

In addition, the statistically significant and high agreement shown by the ICC values between AI model grading and the gold standard grading further supported the model’s favorable performance (Table 1). For nuclear cataract grading in the internal and external datasets, the ICC values were 0.962 and 0.796, respectively, whereas for cortical cataract grading in the internal and external datasets, the ICC values were 0.780 and 0.360, respectively.

The diagnostic capability was further evaluated using the following indices. The accuracy, sensitivity, and specificity of the AI cataract grading were presented, and all showed appreciated results regarding the performance of both nuclear and cortical cataracts (Table 1). Additionally, AI cataract grading for nuclear cataract had an AUC value of 0.983 for the internal dataset (95% CI: 0.965–1.000; *p* < 0.001) and 0.885 for the external dataset (95% CI: 0.788–0.982; *p* < 0.001) (Table 1; Figure 1C), while AI cataract grading for cortical cataract had an AUC value of 0.902 for the internal dataset (95% CI: 0.825–0.978; *p* < 0.001) and 0.984 for the external dataset (95% CI: 0.951–1.000; *p* < 0.001) (Table 1; Figure 1C).

### Performance of postoperative visual acuity prediction

The performances of the postoperative visual acuity prediction in the internal (n = 107) and external (n = 55) datasets were evaluated (Table 2). The model produced more consistent predictions in the internal dataset than in the external dataset, with MAE values of 0.1560 and 0.3057 logMAR and RMSE values of 0.2284 and 0.3922 logMAR for the internal and external datasets, respectively.

**TABLE 2:** Performance of the AI model on the postoperative visual acuity prediction in the internal (n = 107) and external datasets (n = 55).

Parameters	Internal (n = 107)	External (n = 55)
MAE	0.1560	0.3057
RMSE	0.2284	0.3922
Sensitivity in each VA group		
<0.30 logMAR (Snellen 6/12 or higher)	81.61% (71/87)	69.23% (18/26)
≥0.30 logMAR (Snellen 6/12 and lower	55.00% (11/20)	86.21% (25/29)
Precision in each VA group		
<0.30 logMAR (Snellen 6/12 or higher)	87.65% (71/81)	81.82% (18/22)
≥0.30 logMAR (Snellen 6/12 and lower	42.31% (11/26)	75.76% (25/33)

MAE, mean absolute error; RMSE, root mean square error; sensitivity = number of correctly predicted eyes with VA < 0.30 logMAR (or ≥0.30 logMAR)/overall number of eyes having actual VA < 0.30 logMAR (or ≥0.30 logMAR); precision = number of correctly predicted eyes with VA < 0.30 logMAR (or ≥0.30 logMAR)/overall number of eyes having predicted VA < 0.30 logMAR (or ≥0.30 logMAR).

**FIGURE 3:**
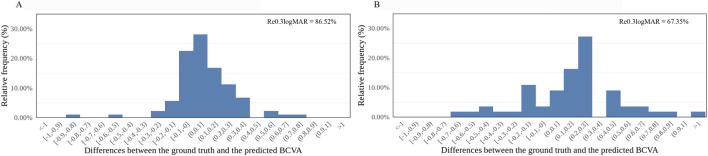
Distribution of the differences between the AI-predicted and actual BCVA. **(A)** The distribution of the differences in the internal dataset (n = 107). **(B)** The distribution of the differences in the external dataset (n = 55). All values are shown in logMAR units. The vertical axis indicates the relative frequency of each BCVA delta value. BCVA, best-corrected visual acuity; logMAR, logarithm of the minimum angle of resolution; Re_0.30logMAR_, the percentage of BCVA prediction errors within ±0.30 logMAR.

In the internal dataset, the sensitivity of this model reached 81.61% (71/87) and 55.00% (11/20) in the good and poor VA groups, respectively. In the external dataset, the sensitivity of this model was 69.23% (18/26) and 86.21% (25/29) in the good and poor VA groups, respectively. In the internal dataset, precision was 87.65% (71/81) and 42.31% (11/26) in the good and poor VA groups, respectively, while in the external dataset, the precision of this model was 81.82% (18/22) and 75.76% (25/33) in the good and poor VA groups, respectively. Differences between the predicted BCVA and the ground truth based on the internal and external datasets are shown in bar charts (Figures 3A,B). The percentages of prediction errors within ±0.30 logMAR were 86.52% using the internal test dataset and 67.35% using the external test dataset.

### Highly myopic cataract surgical decision-making performance

The AI model’s cataract surgical decision-making, along with the gold standard’s and every doctor’s cataract surgical decision-making on all highly myopic eyes, is shown in Table 3.

**TABLE 3:** Surgical decision consistency of the AI model and doctors (senior, junior, and resident) compared to the gold standard (internal/external validation).

Groups	Internal					External				
	Standard	AI	Doctor			Standard	AI	Doctor		
			Senior	Junior	Resident			Senior	Junior	Resident
Surgical decision^a^										
0	1	1	8	8	0	15	7	17	17	6
1	95	96	87	87	91	38	44	33	33	43
2	5	2	6	6	9	0	0	3	3	0
3	6	8	6	6	7	2	4	2	2	6
Consistency (%)										
All decision-making (0, 1, 2, 3)	-	96.26% (103/107)	89.72% (96/107)	89.72% (96/107)	86.92% (93/107)	-	81.82% (45/55)	81.82% (45/55)	81.82% (45/55)	70.91% (39/55)
Special decision-making (0, 2, 3)	-	75.00% (9/12)	83.33% (10/12)	83.33% (10/12)	33.33% (4/12)	-	52.94% (9/17)	76.47% (13/17)	76.47% (13/17)	29.41% (5/17)

^a^ Cataract surgical decisions were categorized as follows: 0 (surgery not advised), 1 (cataract surgery recommended), 2 (retinal surgery recommended), and 3 (combined cataract–retinal surgery recommended).

Performances of AI model and doctors (senior, junior, and resident) were evaluated with consistency compared to the gold standard in internal and external datasets. Consistencies of all decision-making (0, 1, 2, and 3) and decision-making (0, 2, and 3) on special cases were both calculated. A total of 107 eyes were included in the internal datasets, among which 12 eyes required no surgery, retinal surgery, or combined retinal and cataract surgery, whereas 55 eyes were included in the external datasets, among which 17 eyes required no surgery, retinal surgery, or combined retinal surgery. AI, artificial intelligence; consistency = the proportion of correctly predicted surgical decisions of eyes compared to the gold standard.

The performances were evaluated using the internal (n = 107) and external datasets (n = 55). Consistency values were calculated between the AI-based highly myopic cataract decision-making model and the standard, while values were also calculated between doctors of varying experience levels (senior doctor, junior doctor, and resident) and the gold standard (Table 3). The results showed the consistency of 96.26% (103/107) for the AI highly myopic cataract decision-making model in the internal dataset, higher than the consistency of all doctors, including 89.72% (96/107) for the senior doctor, 89.72% (96/107) for the junior doctor, and 86.92% (93/107) for the resident among all eyes. In the external dataset, the consistency of the AI-based decision-making model for highly myopic cataracts was 81.82% (45/55), superior or equal to the consistency of all doctors, specifically 81.82% (45/55) for the senior doctor, 81.82% (45/55) for the junior doctor, and 70.91% (39/55) for the resident among all eyes. This result indicated good performance of the AI model in highly myopic cataract surgical decision-making.

To evaluate the AI model’s performance on special cataract cases, we further calculated the consistency of the AI model and doctors’ decisions compared to the gold standard among special cases in the internal (n = 12) and external datasets (n = 17) (Table 3). The consistencies of the AI model, senior doctor, junior doctor, and resident were 75.00% (9/12), 83.33% (10/12), 83.33% (10/12), and 33.33% (4/12), respectively, in the internal dataset, while in the external dataset, the consistencies of the AI model, senior doctor, junior doctor, and resident were 52.94% (9/17), 76.47% (13/17), and 29.41% (5/17), respectively. Overall, for special cases, the consistency of the AI model was lower than that of senior and junior doctors but significantly higher than that of the resident. These results showed that this AI model’s capability for surgical decision-making in highly myopic cataract cases is less reliable than that of experienced professional doctors. However, this AI model can assist less-experienced doctors, such as the residents, in making highly myopic surgical decisions.

The Kappa values between the AI model, all doctors, and the gold standard were also calculated (Table 4). We first evaluated the decision-making performances of all eyes. In the internal dataset (n = 107), the Kappa values between the AI model and the gold standard were 0.811 (*p* < 0.001), while doctors’ Kappa values in the internal dataset were 0.622 (*p* < 0.001) for the senior doctor, 0.622 (*p* < 0.001) for the junior doctor, and 0.449 (*p* < 0.001) for the resident. In the external dataset (n = 55), the Kappa values between the AI model and the gold standard were 0.556 (*p* < 0.001), while doctors’ Kappa values in the internal dataset were 0.636 (*p* < 0.001) for the senior doctor, 0.636 (*p* < 0.001) for the junior doctor, and 0.317 (*p* < 0.001) for the resident.

**TABLE 4:** Kappa values for highly myopic surgical decision-making performances of the AI model and doctors (senior, junior, and resident) in the internal (n = 107) and external datasets (n = 55).

Groups	Internal		External	
	Kappa^a^	*p*-value	Kappa^a^	*p*-value
AI	0.811	<0.001	0.556	<0.001
Doctor				
Senior	0.622	<0.001	0.636	<0.001
Junior	0.622	<0.001	0.636	<0.001
Resident	0.449	<0.001	0.317	<0.001

^a^ Kappa values were compared to the gold standard.

AI, artificial intelligence.

Additionally, we evaluated the performance of the AI model in making surgical decisions for highly myopic cataract special cases to further assess its effectiveness (Table 5). In the internal dataset (n = 12), the Kappa value between the AI model and the gold standard was 0.609 (*p* < 0.001), while doctors’ Kappa values in the internal dataset were 0.730 (*p* < 0.001) for the senior doctor, 0.730 (*p* < 0.001) for the junior doctor, and −0.103 (*p* = 0.596) for the resident. In the external dataset (n = 17), the Kappa value between the AI model and the gold standard was 0.244 (*p* = 0.003), while doctors’ Kappa values in the internal dataset were 0.139 (*p* = 0.201) for the senior doctor, 0.139 (*p* = 0.201) for the junior doctor, and 0.019 (*p* = 0.793) for the resident.

**TABLE 5:** Kappa values for highly myopic surgical decision-making performance on special cases of the AI cataract decision-making model and doctors (senior, junior, and resident) in the internal and external datasets.

Groups	Internal		External	
	Kappa^a^	*p*-value	Kappa^a^	*p*-value
AI	0.609	<0.001	0.244	0.003
Doctor				
Senior	0.730	<0.001	0.139	0.201
Junior	0.730	<0.001	0.139	0.201
Resident	−0.103	0.596	0.019	0.793

^a^ Kappa values were compared to the gold standard.

The special cases were defined as eyes that required no surgery, retinal surgery, or combined retinal and cataract surgery.

AI, artificial intelligence.

**FIGURE 4:**
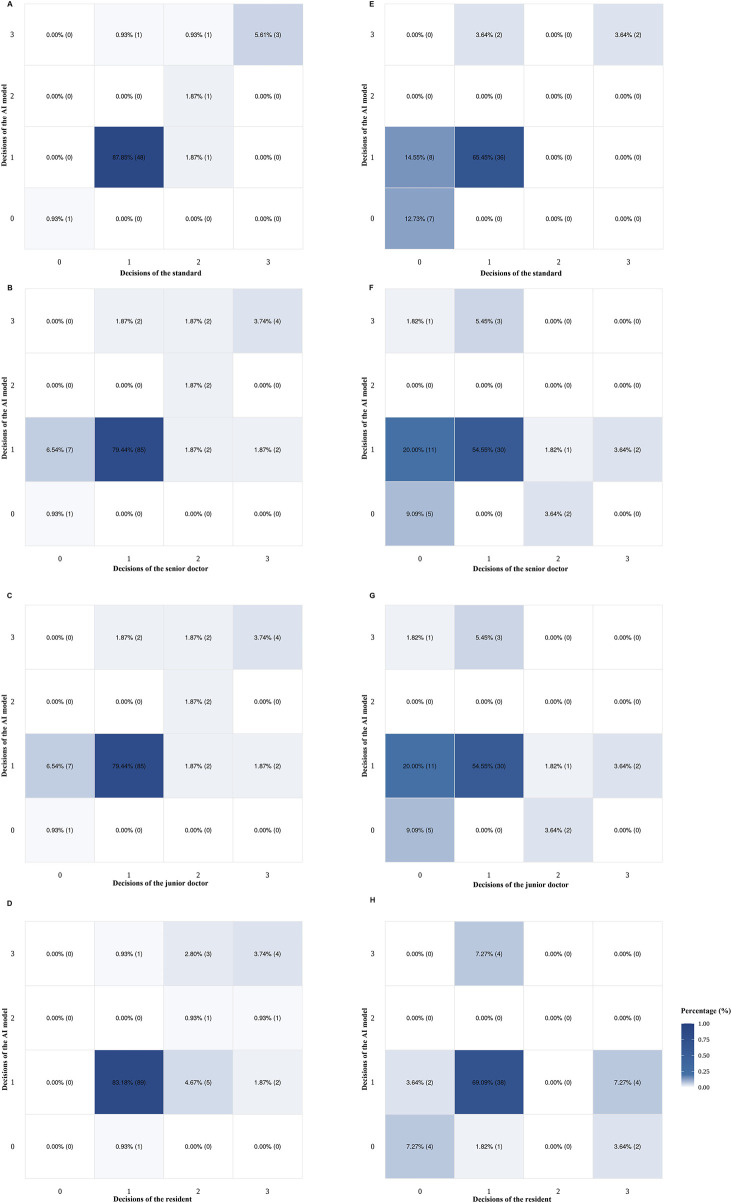
Heatmaps of doctors (senior, junior, and resident) and the AI model for highly myopic cataract surgical decision-making, based on the internal (n =107) and external (n = 55) datasets. **(A)** AI and the gold standard using the internal dataset. **(B)** AI and senior doctors using the internal dataset. **(C)** AI and junior doctors using the internal dataset. **(D)** AI and resident using the internal dataset. **(E)** AI and the gold standard using the external dataset. **(F)** AI and senior doctors using the external dataset. **(G)** AI and junior doctors using the external dataset. **(H)** AI and the resident using the external dataset. AI, artificial intelligence.

Heatmaps illustrating the AI model, the gold standard, and doctors of varying expertise levels (senior doctor, junior doctor, and resident) in highly myopic cataract surgical decision-making are shown in Figure 4. Based on the internal dataset, the AI model matched well with the standard’s decision and doctors’ decisions (Figures 4A–D). However, the AI model’s surgical decision-making performance for special cases in the external dataset was not comparable to that observed in the internal dataset (Figures 4E–H).

## Discussion

Highly myopic cataracts are usually associated with complex fundus pathologies, which may coexist with various vision-threatening conditions, such as epiretinal traction, macular retinoschisis, retinal thickening, foveal retinoschisis, foveal retinal detachment, and CNV (Takano and Kishi, 1999; Baba et al., 2003; Panozzo and Mercanti, 2004; Cheung et al., 2017; Yao et al., 2021). This complexity poses significant challenges to the experience of doctors and primary hospitals that may lack the capability to make accurate surgical decisions independently. Consequently, patients tend to concentrate in tertiary hospitals, exacerbating the uneven distribution of medical resources. To address this issue, our study aimed to develop a deep learning AI model that integrates an automated cataract grading model and a preoperative visual acuity prediction model (Wei et al., 2021; Lu et al., 2022) to assist in surgical decision-making for highly myopic cataracts. In this research, we established a highly myopic cataract decision-making model capable of providing four types of surgical recommendations, namely, no surgery recommended, cataract surgery recommended, retinal surgery recommended, and combined retinal–cataract surgery recommended. The model achieved consistency rates of 96.26% and 81.82% in internal and external datasets, respectively, compared to the gold standard, demonstrated superior consistency over doctors at all levels, and outperformed the resident in complex surgical decision-making on special cases.

Our AI model is based on both an automated cataract grading model and a preoperative visual acuity prediction model as cataract grading and fundus pathology assessment are critically important for surgical decision-making in highly myopic cataracts (Wang et al., 2025; Zhou et al., 2025). Our model exhibits distinct advantages in automated cataract grading, resulting from its foundation in the LOCS III gold standard (Tsao et al., 2022; Wang et al., 2024), which enables precise and consistent classification of cataract subtypes, thereby enhancing surgical decision-making performance (Lu et al., 2022). Simultaneously, our model’s fundus pathology evaluation leverages OCT imaging, which can offer superior details compared to conventional fundus photography-based AI models (Wei et al., 2021; Grzybowski et al., 2024). For instance, the OCT images allow our model to predict postoperative visual acuity, a key factor in surgical decision-making (Wang et al., 2023). In addition, AI models based on OCT images can also diagnose macular diseases (You et al., 2021; Antaki et al., 2024; Feo et al., 2024; Gao et al., 2024). The prediction of postoperative visual acuity demonstrated promising accuracy both in our current study and prior research (Wei et al., 2021). The combined strengths of cataract grading and the OCT-based fundus pathology analysis provide a robust foundation for reliable surgical decision-making in complex highly myopic cataract cases.

We evaluated the AI model’s ability to predict nuclear and cortical cataract grading. The results showed that, for nuclear cataract grading, 99.07% and 87.27% of gradings had absolute prediction errors of ≤1.0 in the internal and external datasets, respectively, while for cortical cataract grading, 88.79% and 61.82% of gradings had absolute prediction errors of ≤1.0 in the internal and external datasets, respectively. Compared to the prior study, our model demonstrated comparable performance in nuclear cataract grading (Lu et al., 2022), suggesting stable classification capabilities for nuclear cataracts. However, the model’s accuracy in cortical cataract grading was lower than that reported in previous work (Lu et al., 2022), particularly in the external dataset. This discrepancy in performance may result from variability under imaging conditions, such as differences in photographic lighting and equipment specifications. To enhance grading consistency, standardized training for slit-lamp photographers may be required prior to using our model in clinical practices.

Additionally, we evaluated AI’s ability to predict the postoperative visual acuity. The MAE values were 0.1560 and 0.3057 logMAR in the internal and external datasets, respectively. In addition, the sensitivity of the AI model was 81.61% and 55.00% in the good and poor VA groups of the internal dataset, respectively, while the sensitivity of the AI model was 69.23% and 86.21% in the good and poor VA groups of the external dataset, respectively. The precision of the AI model was 87.65% and 42.31% in the good and poor VA groups of the internal dataset, respectively, while the precision of the AI model was 81.82% and 75.76% in the good and poor VA groups of the external dataset, respectively. The sensitivity and precision of our model were comparable to those reported in our previous study, showing the stability of our model for OCT-based preoperative visual acuity prediction (Wei et al., 2021). Therefore, it may be applied to assist in surgical decision-making for highly myopic cataracts.

This AI model for highly myopic cataract surgical decision-making demonstrated high consistency with the gold standard—96.26% and 81.82% in internal and external datasets, respectively—exceeding the performance of doctors at all levels. However, for special cases, the performance of this AI model was worse than that of the senior and junior doctors in the analysis of consistency. This result might be related to the insufficient prediction ability for postoperative visual acuity, resulting in an inaccurate decision of whether complex cases should undergo retinal surgery. In addition, a potential limitation of our two prior models is the lack of complex cases in their test datasets. As all data were derived from cataract surgery departments in two hospitals, the limited sample size may introduce random variability. Future validation using larger datasets, specifically those enriched with complex cases, is needed to further strengthen the stability of our model. It is worth mentioning that other deep learning models used preoperative clinical information and color fundus photography (CFP) to predict postoperative visual acuity for cataracts (Yang et al., 2025). Compared to our model, CFP demonstrates inferior visualization of retinal cross-sectional stratification, and severe cataract opacity may significantly compromise CFP image quality. In contrast, OCT provides high-resolution tomographic imaging of retinal layers, enabling precise machine learning (Chen et al., 2023; Ma et al., 2024; Murueta-Goyena et al., 2025). Particularly, in highly myopic cataract cases, OCT better reflects pathological changes in the retina, thus enhancing predictive accuracy. However, in the Kappa analysis, the AI model achieved a higher Kappa value for special cases in the external dataset than all doctors, demonstrating its capability in surgical decision-making for special cases. Some controversies between the analysis of consistency and the Kappa value are possibly attributed to the lack of special cases in our dataset. Overall, the surgical decision-making ability of this AI model was similar to that of the senior and junior doctors and higher than that of the resident, which can assist cataract surgical decision-making of highly myopic eyes in actual clinical practices.

In addition to the internal database, this study also used the external dataset as a separate test subset, which was beneficial for testing the robustness of the AI performance. Generally, in both internal and external datasets, the highly myopic cataract decision-making of our AI model was similar to that of the senior and junior doctors, yet we also found that the performance of AI on the external test dataset was not as promising as that on the internal dataset. The observed differences between the internal and external datasets might result from variations in the OCT equipment used for image acquisition in the external dataset compared to that used during model training. Although we implemented standardized photo preprocessing throughout the entire research process to minimize potential differences, the existence of relevant restrictions inevitably introduced bias. Furthermore, as there is currently a lack of consensus and standardized guidelines regarding the decision-making criteria for highly myopic cataract surgery in clinical practice, we acknowledge this as a limitation of our study, in which experienced specialists were used as the gold standard. In future research, we aim to address the issue of insufficient universality by incorporating multicenter data into the AI training process and further improving the decision accuracy of the AI model. At present, due to the high difficulty of diagnosing and treating high-myopia cataracts in clinical practice, the surgical decisions made through deep learning models may still provide valuable references for preoperative communication and surgical treatment of this special population (Tognetto et al., 2022).

## Conclusion

In summary, based on automatic grading models for different types of cataracts and postoperative visual acuity prediction algorithms, we are pioneering the development of a deep learning prediction model specifically designed for highly myopic cataract surgical decision-making. This model can provide logical decision-making strategies for the surgical treatment of patients with highly myopic cataracts. Our model will help provide a reliable reference for surgical decision-making in highly myopic cataract patients, laying the foundation for the development of an independent AI model in the future.

## Data Availability

The data analyzed in this study are subject to the following licenses/restrictions: the raw data supporting this study’s conclusions are accessible from the corresponding authors upon justified request, subject to compliance with ethical protocols. Requests to access these datasets should be directed to zhuxiangjia1982@126.com.

## Supplementary material

SUPPLEMENTARY FIGURE 1 Flowchart illustrating the logic of the AI-based highly myopic cataract surgical decision-making model. This AI surgical decision-making model processes input data through a sequential five-step workflow to generate surgical recommendations: preoperative visual acuity assessment, postoperative visual improvement evaluation, automated cataract grading, OCT image analysis, and axial length measurement. The surgical decisions can be categorized into seven detailed groups: surgery not recommended, surgery not recommended (consult a doctor for details), cataract surgery recommended, cataract surgery recommended (consult a doctor for details), retinal surgery recommended, combined cataract and retinal surgery recommended, and combined cataract and retinal surgery recommended (consult a doctor for details). C, cortical cataract; N, nuclear cataract; P, posterior subscapular cataract; CNV, choroidal neovascularization; AL, axial length.
